# Crystal structure of ethyl 2-(4-chlorophenyl)-3-cyclopentyl-4-oxo-1-propyl­imidazolidine-5-carboxylate

**DOI:** 10.1107/S2056989015015364

**Published:** 2015-08-22

**Authors:** Mohamed Ali Tabarki, Youssef Ben Smida, Abderrahmen Guesmi, Rafâa Besbes

**Affiliations:** a Laboratoire de Chimie Analytique et Electrochimie, Faculté des Sciences de Tunis, Université de Tunis El Manar, 2092 Manar II, Tunis, Tunisia; b Laboratoire de Matériaux et Cristallochimie, Faculté des Sciences de Tunis, Université de Tunis El Manar, 2092 Manar II Tunis, Tunisia; c Institut Préparatoire aux Etudes d’Ingénieurs d’El Manar, El Manar II, 2092 Tunis, Tunisia

**Keywords:** crystal structure, synthesis, aziridine rearrangement, C—H⋯O and C—H⋯O inter­actions

## Abstract

The title compound, C_20_H_27_ClN_2_O_3_, was obtained *via* an original synthesis method. The central heterocyclic ring adopts a shallow envelope conformation, with the N atom bearing the cyclo­pentane ring as the flap [deviation from the other atoms = 0.442 (2) Å]. The cyclo­pentane ring adopts a twisted conformation about one of the C_N_—C bonds: the exocyclic C—N bond adopts an equatorial orientation. The dihedral angles between the central ring (all atoms) and the pendant five- and six-membered rings are 10.3 (2) and 87.76 (14)°, respectively. In the crystal, C—H⋯O inter­actions link the mol­ecules into [011] chains. A weak C—H⋯Cl inter­action links the chains into (100) sheets. A mechanism for the cyclization reaction is proposed.

## Related literature   

For background to the biological properties of imidazolidin-4-one rings, see: Chambel *et al.* (2006 ▸); Vale *et al.* (2008*a* ▸,*b* ▸,*c* ▸); Gomes *et al.* (2004 ▸); Araujo *et al.* (2005 ▸); Qin *et al.* (2009 ▸). For imidazolidin-4-one rings in Diels–Alder reactions, see: Lin *et al.* (2013 ▸). For the synthesis and mechanistic studies, see: Gomes *et al.* (2006 ▸); Zhang *et al.* (2008 ▸).
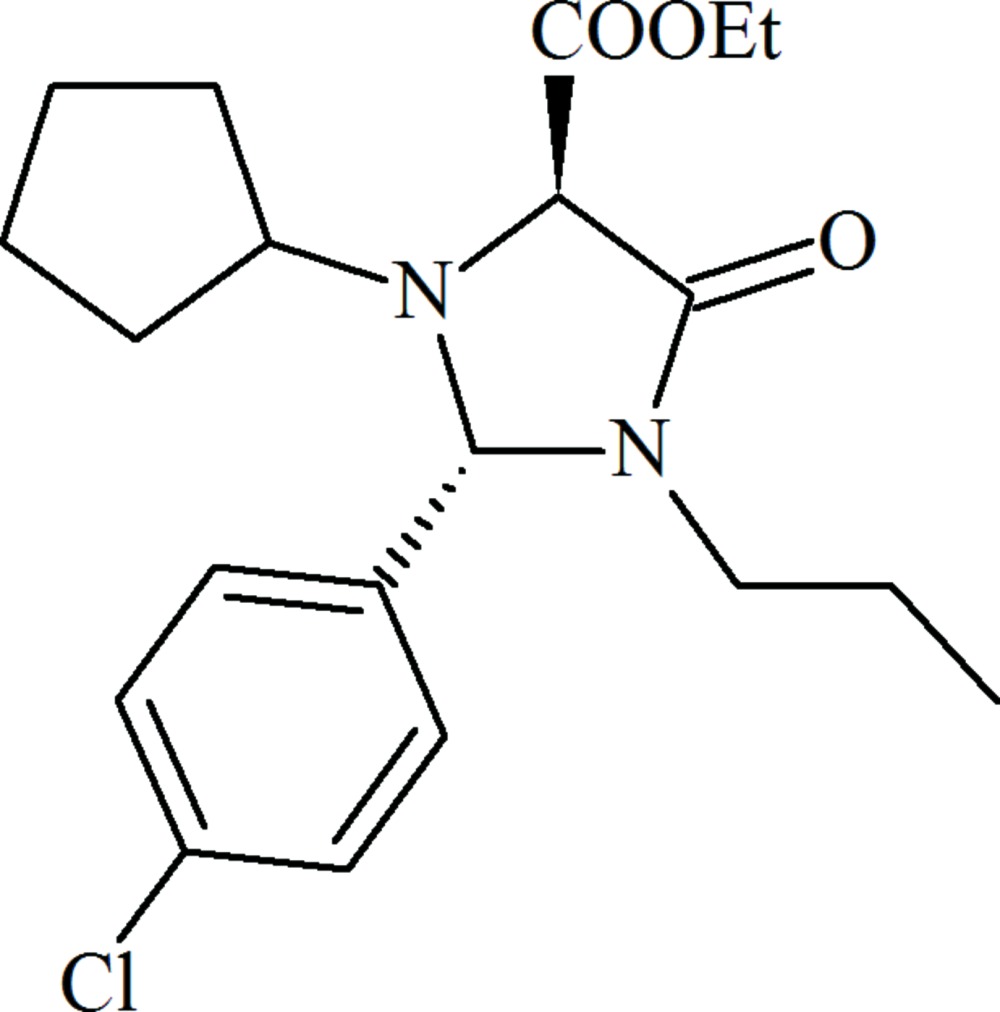


## Experimental   

### Crystal data  

C_20_H_27_ClN_2_O_3_
*M* *_r_* = 378.89
Triclinic, 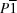

*a* = 9.083 (7) Å
*b* = 11.201 (6) Å
*c* = 11.846 (6) Å
α = 117.75 (4)°
β = 90.49 (5)°
γ = 104.08 (6)°
*V* = 1024.1 (11) Å^3^
*Z* = 2
Mo *K*α radiation
μ = 0.21 mm^−1^
*T* = 298 K
0.4 × 0.3 × 0.2 mm

### Data collection  

Enraf–Nonius CAD-4 diffractometer
6270 measured reflections
4439 independent reflections
2533 reflections with *I* > 2σ(*I*)
*R*_int_ = 0.024
2 standard reflections every 120 reflections intensity decay: 4%

### Refinement  

*R*[*F* ^2^ > 2σ(*F* ^2^)] = 0.057
*wR*(*F* ^2^) = 0.180
*S* = 0.99
4439 reflections
295 parameters
H atoms treated by a mixture of independent and constrained refinement
Δρ_max_ = 0.31 e Å^−3^
Δρ_min_ = −0.19 e Å^−3^

### 

Data collection: *CAD-4 EXPRESS* (Duisenberg, 1992 ▸; Macíček & Yordanov, 1992 ▸); cell refinement: *CAD-4 EXPRESS*; data reduction: *XCAD4* (Harms & Wocadlo, 1995 ▸); program(s) used to solve structure: *SHELXS97* (Sheldrick, 2008 ▸); program(s) used to refine structure: *SHELXL97* (Sheldrick, 2008 ▸); molecular graphics: *DIAMOND* (Brandenburg & Putz, 2005 ▸); software used to prepare material for publication: *WinGX* (Farrugia, 2012 ▸) and *publCIF* (Westrip, 2010 ▸).

## Supplementary Material

Crystal structure: contains datablock(s) I, New_Global_Publ_Block. DOI: 10.1107/S2056989015015364/hb7486sup1.cif

Structure factors: contains datablock(s) I. DOI: 10.1107/S2056989015015364/hb7486Isup2.hkl

Supporting information file. DOI: 10.1107/S2056989015015364/hb7486Isup3.cml

**Fig. 1.**
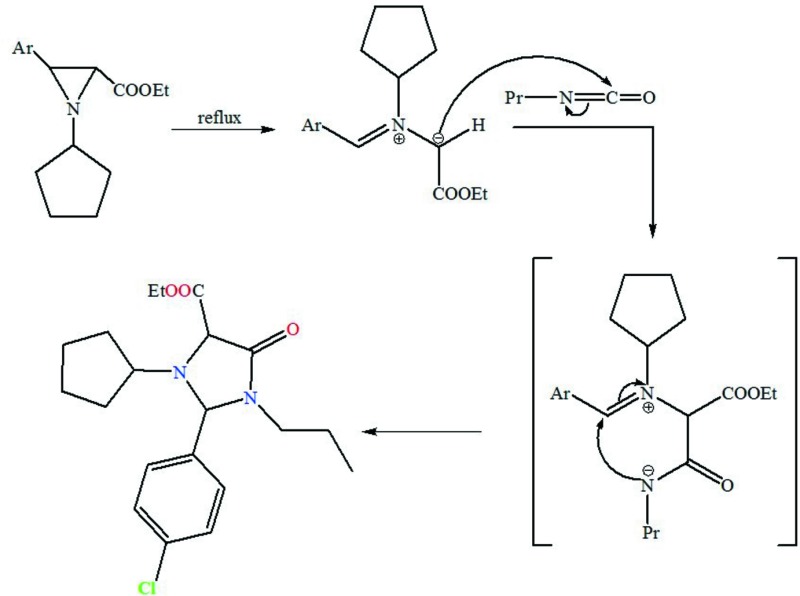
Synthesis protocol of C20H27ClN2O3.

20 27 2 3 . DOI: 10.1107/S2056989015015364/hb7486fig1.tif
Synthesis protocol of C_20_H_27_ClN_2_O_3_.

**Fig. 2.**
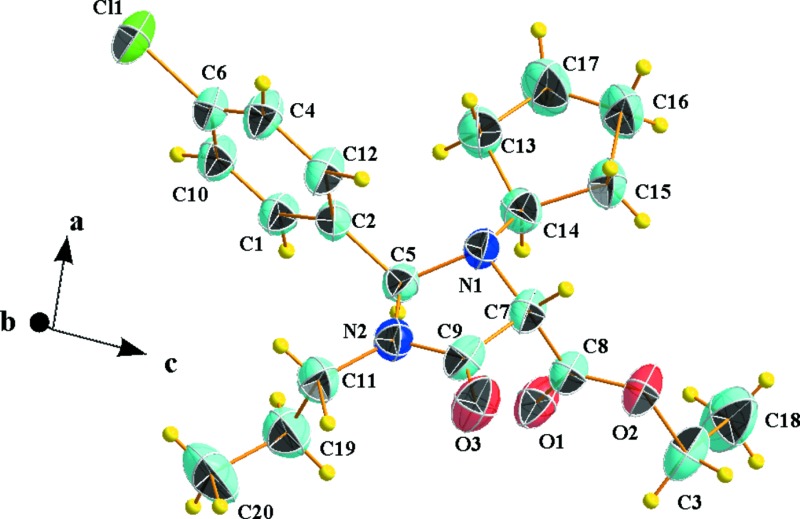
Perspective view of the title compound showing 50% displacement ellipsoids.

. DOI: 10.1107/S2056989015015364/hb7486fig2.tif
Perspective view of the title compound showing 50% displacement ellipsoids.

**Fig. 3.**
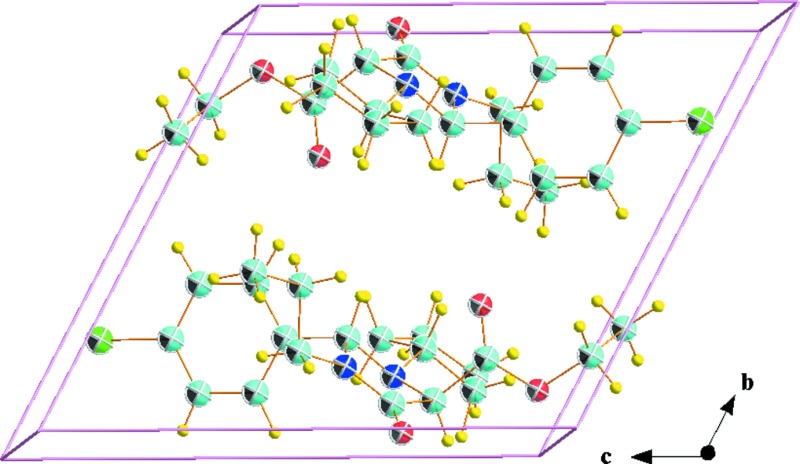
Unit cell projection of C20H27ClN2O3 showing two molecules per cell.

20 27 2 3 . DOI: 10.1107/S2056989015015364/hb7486fig3.tif
Unit cell projection of C_20_H_27_ClN_2_O_3_ showing two mol­ecules per cell.

CCDC reference: 1419261

Additional supporting information:  crystallographic information; 3D view; checkCIF report

## Figures and Tables

**Table 1:** Hydrogen-bond geometry (, )

*D*H*A*	*D*H	H*A*	*D* *A*	*D*H*A*
C10H2O1^i^	1.00(2)	2.50(3)	3.454(4)	160(2)
C3H12O3^ii^	0.99(4)	2.59(4)	3.439(5)	143(3)
C16H16*B*Cl1^iii^	0.97	2.80	3.662(6)	148

Symmetry codes: (i) 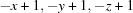; (ii) 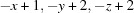; (iii) 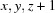.

## supplementary crystallographic information

### S1. comment

The imidazolidin-4-one rings are of major inter­est and constitute a very important class of heterocyclic compounds because of their presence in several biologically active synthetic products (Chambel *et al.* 2006; Vale *et al.* 2008a) and their use as high anti­malarial drugs (Vale *et al.* 2008b&c). These products exhibit also anti­bacterial activity (Gomes *et al.* 2004; Araujo *et al.* 2005) and inhibit binding of VCAM-1 to VLA-4 (Qin *et al.* 2009). On the other hand, imidazolidnione was used as organocatalyst for Diels-Alder reactions (Lin *et al.*2013).

In the present work we have developed an efficient strategy for the synthesis of 1-cyclo­penty-2-para­chloro­phenyl-3-propyl-5-eth­oxy­carbonyl­imidazolidin-4-one (I) (Fig.1) via ring expansion of aziridine-2-carboxyl­ate upon reaction with propyl­iso­cyanate. It should be mentioned that in a similar protocol, Gomes *et al.* (2006) report that aziridines rearrange under the effect of heating or radiation and transform into azomethines. The latter reacts subsequently on various electrophiles systems.

A result similar to one described by Zhang *et al.*(2008), but the authors did not explain the formation of the compounds obtained. To explain the formation of the imidazolidin-4-one we based on work that was performed by Gomes *et al.* (2006) and in which the authors suggest that aziridines rearrange under the effect of heating or irradiation and become an azomethine. The latter reacts subsequently on various electrophile systems. In our case, the attack of the iso­cyanate by the carbanion of azomethine, formed upon the refluxing in toluene aziridine, adequately explains obtaining imidazolidin-4-one after cyclization of the inter­mediate formed.

### S2.1. Synthesis and crystallization

To a solution of ethyl 3-(4-chloro­phenyl)-1-cyclo­pentyl­aziridine-2-carboxyl­ate (2.20 mmol) in toluene (10 ml) under nitro­gen atmosphere, were added n-Propyl­iso­cyanate (2.64 mmol). The mixture was refluxed during 20 hours. After completeness of the reaction, the mixture was concentrated under reduced pressure and the residue was purified by silica gel column chromatography using a mixture of n-hexane / EtOAc (5:5) as eluent to afford colourless prisms of the studied compound.

### S2.2. Refinement

Hydrogen atoms were treated by a mixture of independent and constrained refinement. In fact hydrogen atoms from H1 to H15 were located in the difference Fourier Map. The others H atoms were located geometrically and refined using a riding model.

### Crystal data

**Table:**

C_20_H_27_ClN_2_O_3_	*Z* = 2
*M**_r_* = 378.89	*F*(000) = 404
Triclinic, *P*1	*D*_x_ = 1.229 Mg m^−^^3^
Hall symbol: -P 1	Mo *K*α radiation, λ = 0.71073 Å
*a* = 9.083 (7) Å	Cell parameters from 25 reflections
*b* = 11.201 (6) Å	θ = 10–15°
*c* = 11.846 (6) Å	µ = 0.21 mm^−^^1^
α = 117.75 (4)°	*T* = 298 K
β = 90.49 (5)°	Prism, colorless
γ = 104.08 (6)°	0.4 × 0.3 × 0.2 mm
*V* = 1024.1 (11) Å^3^	

### Data collection

**Table:**

Enraf–Nonius CAD-4 diffractometer	*R*_int_ = 0.024
Radiation source: fine-focus sealed tube	θ_max_ = 27.0°, θ_min_ = 2.1°
Graphite monochromator	*h* = −11→3
ω/2θ scans	*k* = −14→14
6270 measured reflections	*l* = −15→15
4439 independent reflections	2 standard reflections every 120 reflections
2533 reflections with *I* > 2σ(*I*)	intensity decay: 4%

### Refinement

**Table:**

Refinement on *F*^2^	Primary atom site location: structure-invariant direct methods
Least-squares matrix: full	Secondary atom site location: difference Fourier map
*R*[*F*^2^ > 2σ(*F*^2^)] = 0.057	Hydrogen site location: inferred from neighbouring sites
*wR*(*F*^2^) = 0.180	H atoms treated by a mixture of independent and constrained refinement
*S* = 0.99	*w* = 1/[σ^2^(*F*_o_^2^) + (0.1064*P*)^2^ + 0.0609*P*] where *P* = (*F*_o_^2^ + 2*F*_c_^2^)/3
4439 reflections	(Δ/σ)_max_ = 0.043
295 parameters	Δρ_max_ = 0.31 e Å^−^^3^
0 restraints	Δρ_min_ = −0.19 e Å^−^^3^

### Special details

**Table:**

Geometry. All e.s.d.'s (except the e.s.d. in the dihedral angle between two l.s. planes) are estimated using the full covariance matrix. The cell e.s.d.'s are taken into account individually in the estimation of e.s.d.'s in distances, angles and torsion angles; correlations between e.s.d.'s in cell parameters are only used when they are defined by crystal symmetry. An approximate (isotropic) treatment of cell e.s.d.'s is used for estimating e.s.d.'s involving l.s. planes.
Refinement. Refinement of *F*^2^ against ALL reflections. The weighted *R*-factor *wR* and goodness of fit *S* are based on *F*^2^, conventional *R*-factors *R* are based on *F*, with *F* set to zero for negative *F*^2^. The threshold expression of *F*^2^ > σ(*F*^2^) is used only for calculating *R*-factors(gt) *etc*. and is not relevant to the choice of reflections for refinement. *R*-factors based on *F*^2^ are statistically about twice as large as those based on *F*, and *R*- factors based on ALL data will be even larger.

### Fractional atomic coordinates and isotropic or equivalent isotropic displacement parameters (Å^2^)

**Table:**

	*x*	*y*	*z*	*U*_iso_*/*U*_eq_	
Cl1	1.01028 (10)	0.71959 (8)	0.06769 (7)	0.0791 (3)	
O1	0.5260 (2)	0.67479 (19)	0.72389 (18)	0.0698 (6)	
O2	0.5879 (2)	0.87245 (18)	0.91481 (16)	0.0650 (5)	
O3	0.4728 (3)	0.9785 (2)	0.69803 (19)	0.0759 (6)	
N1	0.7774 (2)	0.82492 (19)	0.64046 (17)	0.0461 (5)	
N2	0.5635 (2)	0.8158 (2)	0.53131 (18)	0.0502 (5)	
C1	0.8327 (3)	0.6004 (3)	0.1915 (2)	0.0570 (7)	
C2	0.7634 (3)	0.7335 (2)	0.4011 (2)	0.0441 (5)	
C3	0.4957 (4)	0.7979 (4)	0.9770 (3)	0.0732 (9)	
C4	0.9202 (4)	0.8525 (3)	0.3002 (2)	0.0606 (7)	
C5	0.6794 (3)	0.7399 (2)	0.5138 (2)	0.0442 (5)	
C6	0.9138 (3)	0.7244 (3)	0.1961 (2)	0.0538 (6)	
C7	0.6788 (3)	0.8889 (2)	0.7346 (2)	0.0503 (6)	
C8	0.5893 (3)	0.7974 (3)	0.7891 (2)	0.0502 (6)	
C9	0.5592 (3)	0.9044 (2)	0.6558 (2)	0.0527 (6)	
C10	0.7574 (3)	0.6052 (2)	0.2946 (2)	0.0507 (6)	
C11	0.4526 (4)	0.7868 (3)	0.4255 (3)	0.0635 (7)	
C12	0.8435 (3)	0.8565 (3)	0.4024 (2)	0.0559 (7)	
C13	1.0021 (4)	0.7211 (4)	0.5907 (3)	0.0706 (8)	
C14	0.8731 (3)	0.7538 (3)	0.6745 (2)	0.0555 (6)	
C15	0.9604 (4)	0.8461 (4)	0.8118 (3)	0.0717 (8)	
C16	1.1058 (4)	0.8014 (6)	0.8072 (4)	0.1159 (15)	
H16A	1.1940	0.8836	0.8458	0.139*	
H16B	1.1023	0.7511	0.8556	0.139*	
C17	1.1201 (4)	0.7121 (5)	0.6750 (4)	0.1030 (12)	
H17A	1.1022	0.6159	0.6581	0.124*	
H17B	1.2224	0.7432	0.6574	0.124*	
C18	0.5848 (5)	0.7287 (5)	1.0162 (4)	0.1136 (14)	
H18A	0.5229	0.6808	1.0561	0.170*	
H18B	0.6730	0.7974	1.0764	0.170*	
H18C	0.6176	0.6619	0.9419	0.170*	
C19	0.3480 (4)	0.6381 (3)	0.3616 (3)	0.0785 (9)	
H19A	0.4093	0.5724	0.3275	0.094*	
H19B	0.2937	0.6217	0.4255	0.094*	
C20	0.2353 (5)	0.6120 (6)	0.2556 (4)	0.1300 (17)	
H20A	0.1705	0.5173	0.2180	0.195*	
H20B	0.2886	0.6256	0.1911	0.195*	
H20C	0.1737	0.6762	0.2892	0.195*	
H1	0.626 (2)	0.645 (2)	0.4965 (19)	0.032 (5)*	
H2	0.697 (3)	0.519 (2)	0.297 (2)	0.041 (6)*	
H3	0.734 (3)	0.979 (3)	0.802 (3)	0.067 (8)*	
H4	0.392 (4)	0.738 (4)	0.923 (3)	0.096 (11)*	
H5	0.826 (3)	0.513 (3)	0.115 (3)	0.073 (8)*	
H6	0.972 (3)	0.937 (3)	0.310 (3)	0.073 (9)*	
H7	0.806 (3)	0.668 (3)	0.667 (2)	0.051 (6)*	
H8	0.497 (4)	0.796 (3)	0.365 (3)	0.082 (10)*	
H9	0.952 (4)	0.624 (3)	0.516 (3)	0.091 (10)*	
H10	0.842 (3)	0.951 (3)	0.476 (3)	0.075 (8)*	
H11	0.908 (4)	0.827 (3)	0.868 (3)	0.090 (10)*	
H12	0.470 (4)	0.875 (4)	1.053 (4)	0.098 (11)*	
H13	0.375 (4)	0.834 (4)	0.460 (3)	0.107 (12)*	
H14	1.051 (4)	0.812 (4)	0.585 (3)	0.100 (11)*	
H15	0.982 (4)	0.953 (4)	0.834 (3)	0.091 (10)*	

### Atomic displacement parameters (Å^2^)

**Table:**

	*U*^11^	*U*^22^	*U*^33^	*U*^12^	*U*^13^	*U*^23^
Cl1	0.0968 (6)	0.0929 (6)	0.0563 (4)	0.0280 (5)	0.0331 (4)	0.0418 (4)
O1	0.0915 (14)	0.0523 (11)	0.0569 (11)	0.0042 (10)	0.0224 (10)	0.0269 (9)
O2	0.0894 (14)	0.0616 (11)	0.0461 (9)	0.0212 (10)	0.0243 (9)	0.0274 (8)
O3	0.0993 (16)	0.0773 (13)	0.0715 (13)	0.0525 (12)	0.0327 (11)	0.0386 (11)
N1	0.0511 (11)	0.0486 (11)	0.0392 (9)	0.0109 (9)	0.0068 (8)	0.0231 (8)
N2	0.0523 (12)	0.0537 (11)	0.0478 (11)	0.0174 (9)	0.0079 (9)	0.0259 (9)
C1	0.0669 (17)	0.0512 (14)	0.0442 (13)	0.0164 (13)	0.0117 (12)	0.0160 (12)
C2	0.0476 (13)	0.0434 (12)	0.0386 (11)	0.0092 (10)	0.0040 (10)	0.0195 (10)
C3	0.098 (3)	0.077 (2)	0.0580 (17)	0.034 (2)	0.0333 (18)	0.0390 (16)
C4	0.082 (2)	0.0509 (15)	0.0496 (14)	0.0093 (14)	0.0154 (13)	0.0292 (13)
C5	0.0466 (13)	0.0408 (12)	0.0431 (12)	0.0079 (10)	0.0092 (10)	0.0206 (10)
C6	0.0582 (15)	0.0662 (16)	0.0408 (12)	0.0165 (12)	0.0115 (11)	0.0291 (12)
C7	0.0661 (16)	0.0399 (12)	0.0402 (12)	0.0088 (11)	0.0116 (11)	0.0185 (10)
C8	0.0585 (15)	0.0484 (14)	0.0449 (12)	0.0150 (12)	0.0134 (11)	0.0232 (11)
C9	0.0648 (16)	0.0467 (13)	0.0522 (14)	0.0180 (12)	0.0199 (12)	0.0268 (11)
C10	0.0552 (15)	0.0440 (13)	0.0485 (13)	0.0104 (11)	0.0102 (11)	0.0205 (11)
C11	0.0627 (18)	0.0772 (19)	0.0590 (17)	0.0239 (16)	0.0066 (14)	0.0375 (16)
C12	0.0757 (18)	0.0411 (13)	0.0445 (13)	0.0083 (12)	0.0145 (12)	0.0192 (11)
C13	0.074 (2)	0.081 (2)	0.0568 (17)	0.0360 (18)	0.0077 (15)	0.0262 (17)
C14	0.0581 (16)	0.0553 (15)	0.0593 (15)	0.0118 (13)	0.0038 (13)	0.0346 (13)
C15	0.073 (2)	0.100 (3)	0.0519 (16)	0.0268 (18)	0.0068 (14)	0.0434 (17)
C16	0.080 (3)	0.208 (5)	0.073 (2)	0.058 (3)	0.0089 (19)	0.070 (3)
C17	0.087 (3)	0.151 (3)	0.083 (2)	0.061 (3)	0.008 (2)	0.053 (2)
C18	0.129 (3)	0.156 (4)	0.120 (3)	0.067 (3)	0.041 (3)	0.104 (3)
C19	0.072 (2)	0.084 (2)	0.0719 (19)	0.0244 (17)	0.0029 (16)	0.0309 (17)
C20	0.087 (3)	0.165 (4)	0.110 (3)	0.025 (3)	−0.030 (2)	0.050 (3)

### Geometric parameters (Å, º)

**Table:**

Cl1—C6	1.747 (3)	C11—C19	1.515 (5)
O1—C8	1.197 (3)	C11—H8	0.86 (3)
O2—C8	1.328 (3)	C11—H13	0.96 (4)
O2—C3	1.478 (3)	C12—H10	1.01 (3)
O3—C9	1.222 (3)	C13—C17	1.511 (5)
N1—C7	1.464 (3)	C13—C14	1.543 (4)
N1—C5	1.478 (3)	C13—H9	1.01 (3)
N1—C14	1.478 (3)	C13—H14	1.03 (4)
N2—C9	1.348 (3)	C14—C15	1.535 (4)
N2—C11	1.460 (4)	C14—H7	0.97 (2)
N2—C5	1.466 (3)	C15—C16	1.515 (5)
C1—C6	1.380 (4)	C15—H11	0.90 (3)
C1—C10	1.391 (3)	C15—H15	1.07 (3)
C1—H5	0.96 (3)	C16—C17	1.441 (5)
C2—C12	1.385 (3)	C16—H16A	0.9700
C2—C10	1.390 (3)	C16—H16B	0.9700
C2—C5	1.525 (3)	C17—H17A	0.9700
C3—C18	1.452 (5)	C17—H17B	0.9701
C3—H4	1.01 (4)	C18—H18A	0.9600
C3—H12	0.99 (4)	C18—H18B	0.9600
C4—C6	1.376 (4)	C18—H18C	0.9600
C4—C12	1.390 (4)	C19—C20	1.483 (5)
C4—H6	0.90 (3)	C19—H19A	0.9700
C5—H1	0.97 (2)	C19—H19B	0.9700
C7—C9	1.518 (4)	C20—H20A	0.9600
C7—C8	1.533 (3)	C20—H20B	0.9599
C7—H3	0.95 (3)	C20—H20C	0.9600
C10—H2	1.00 (2)		
			
C8—O2—C3	116.5 (2)	C2—C12—C4	120.8 (2)
C7—N1—C5	106.60 (19)	C2—C12—H10	119.8 (16)
C7—N1—C14	116.08 (19)	C4—C12—H10	119.3 (16)
C5—N1—C14	115.95 (19)	C17—C13—C14	103.6 (3)
C9—N2—C11	123.2 (2)	C17—C13—H9	109.5 (18)
C9—N2—C5	113.4 (2)	C14—C13—H9	103.7 (19)
C11—N2—C5	123.1 (2)	C17—C13—H14	105 (2)
C6—C1—C10	119.3 (2)	C14—C13—H14	105.6 (19)
C6—C1—H5	118.8 (17)	H9—C13—H14	127 (3)
C10—C1—H5	121.8 (17)	N1—C14—C15	112.0 (2)
C12—C2—C10	119.2 (2)	N1—C14—C13	113.7 (2)
C12—C2—C5	120.1 (2)	C15—C14—C13	103.3 (2)
C10—C2—C5	120.7 (2)	N1—C14—H7	107.9 (14)
C18—C3—O2	110.8 (3)	C15—C14—H7	109.7 (14)
C18—C3—H4	117 (2)	C13—C14—H7	110.2 (14)
O2—C3—H4	111 (2)	C16—C15—C14	104.7 (3)
C18—C3—H12	111 (2)	C16—C15—H11	107 (2)
O2—C3—H12	103 (2)	C14—C15—H11	111 (2)
H4—C3—H12	103 (3)	C16—C15—H15	113.1 (18)
C6—C4—C12	119.1 (2)	C14—C15—H15	107.8 (18)
C6—C4—H6	125.9 (19)	H11—C15—H15	113 (3)
C12—C4—H6	115.0 (19)	C17—C16—C15	109.3 (3)
N2—C5—N1	101.47 (18)	C17—C16—H16A	109.8
N2—C5—C2	110.85 (19)	C15—C16—H16A	109.8
N1—C5—C2	113.85 (19)	C17—C16—H16B	109.8
N2—C5—H1	107.4 (12)	C15—C16—H16B	109.8
N1—C5—H1	113.1 (12)	H16A—C16—H16B	108.3
C2—C5—H1	109.7 (12)	C16—C17—C13	107.5 (3)
C4—C6—C1	121.2 (2)	C16—C17—H17A	110.2
C4—C6—Cl1	119.1 (2)	C13—C17—H17A	110.2
C1—C6—Cl1	119.7 (2)	C16—C17—H17B	110.2
N1—C7—C9	103.22 (19)	C13—C17—H17B	110.2
N1—C7—C8	115.3 (2)	H17A—C17—H17B	108.5
C9—C7—C8	105.8 (2)	C3—C18—H18A	109.5
N1—C7—H3	111.6 (16)	C3—C18—H18B	109.5
C9—C7—H3	109.3 (16)	H18A—C18—H18B	109.5
C8—C7—H3	111.0 (16)	C3—C18—H18C	109.5
O1—C8—O2	125.5 (2)	H18A—C18—H18C	109.5
O1—C8—C7	123.1 (2)	H18B—C18—H18C	109.5
O2—C8—C7	111.4 (2)	C20—C19—C11	111.8 (3)
O3—C9—N2	127.3 (3)	C20—C19—H19A	109.3
O3—C9—C7	126.4 (2)	C11—C19—H19A	109.3
N2—C9—C7	106.2 (2)	C20—C19—H19B	109.3
C2—C10—C1	120.4 (2)	C11—C19—H19B	109.3
C2—C10—H2	116.7 (12)	H19A—C19—H19B	107.9
C1—C10—H2	122.9 (12)	C19—C20—H20A	109.5
N2—C11—C19	112.9 (3)	C19—C20—H20B	109.5
N2—C11—H8	112 (2)	H20A—C20—H20B	109.5
C19—C11—H8	106 (2)	C19—C20—H20C	109.5
N2—C11—H13	109 (2)	H20A—C20—H20C	109.5
C19—C11—H13	97 (2)	H20B—C20—H20C	109.5
H8—C11—H13	119 (3)		

### Hydrogen-bond geometry (Å, º)

**Table:**

*D*—H···*A*	*D*—H	H···*A*	*D*···*A*	*D*—H···*A*
C10—H2···O1^i^	1.00 (2)	2.50 (3)	3.454 (4)	160 (2)
C3—H12···O3^ii^	0.99 (4)	2.59 (4)	3.439 (5)	143 (3)
C16—H16*B*···Cl1^iii^	0.97	2.80	3.662 (6)	148

Symmetry codes: (i) −*x*+1, −*y*+1, −*z*+1; (ii) −*x*+1, −*y*+2, −*z*+2; (iii) *x*, *y*, *z*+1.

## References

[bb1] Araújo, M. J., Bom, J., Capela, R., Casimiro, C., Chambel, P., Gomes, P., Iley, J., Lopes, F., Morais, J., Moreira, R., de Oliveira, E., do Rosário, V. & Vale, N. (2005). *J. Med. Chem.* **48**, 888–892. 10.1021/jm0494624 15689174

[bb2] Brandenburg, K. & Putz, H. (2005). *DIAMOND*. Crystal Impact GbR, Bonn, Germany.

[bb3] Chambel, P., Capela, R., Lopes, F., Iley, J., Morais, J., Gouveia, L., Gomes, J. R. B., Gomes, P. & Moreira, R. (2006). *Tetrahedron*, **62**, 9883–9891.

[bb4] Duisenberg, A. J. M. (1992). *J. Appl. Cryst.* **25**, 92–96.

[bb5] Farrugia, L. J. (2012). *J. Appl. Cryst.* **45**, 849–854.

[bb6] Gomes, R., Araújo, M. J., Rodrigues, M., Vale, N., Azevedo, Z., Iley, J., Chambel, P., Morais, J. & Moreira, R. (2004). *Tetrahedron*, **60**, 5551–5562.

[bb7] Gomes, P. J. S., Nunes, C. M., Pais, A. A. C. C., Pinho e Melo, T. M. V. D. & Arnaut, L. G. (2006). *Tetrahedron Lett.* **47**, 5475–5479.

[bb8] Harms, K. & Wocadlo, S. (1995). *XCAD4*. University of Marburg, Germany.

[bb9] Lin, Z., Chen, Z., Yang, G. & Lu, C. (2013). *Catal. Commun.* **35**, 1–5.

[bb10] Macíček, J. & Yordanov, A. (1992). *J. Appl. Cryst.* **25**, 73–80.

[bb11] Qin, L. Y., Cole, A. G., Metzger, A., O’Brien, L., Sun, X., Wu, J., Xu, Y., Xu, K., Zhang, Y. & Henderson, I. (2009). *Tetrahedron Lett.* **50**, 419–422.

[bb12] Sheldrick, G. M. (2008). *Acta Cryst.* A**64**, 112–122. 10.1107/S0108767307043930 18156677

[bb13] Vale, N., Collins, M. S., Gut, J., Ferraz, R., Rosenthal, P. J., Cushion, M. T., Moreira, R. & Gomes, P. (2008*a*). *Bioorg. Med. Chem. Lett.* **18**, 485–488. 10.1016/j.bmcl.2007.11.105 18077165

[bb14] Vale, N., Matos, J., Moreira, R. & Gomes, P. (2008*b*). *Tetrahedron*, **64**, 11144–11149.

[bb15] Vale, N., Moreira, R. & Gomes, P. (2008*c*). *Int. J. Mass Spectrom.* **270**, 81–93.

[bb16] Westrip, S. P. (2010). *J. Appl. Cryst.* **43**, 920–925.

[bb17] Zhang, K., Chopade, P. R. & Louie, J. (2008). *Tetrahedron Lett.* **49**, 4306–4309. 10.1016/j.tetlet.2008.04.121 PMC2515493 19122764

