# Supplementary figures and images for: Crystal structure of ethyl 2-(4-chlorophenyl)-3-cyclopentyl-4-oxo-1-propyl­imidazolidine-5-carboxylate
Source: Acta Crystallogr E Crystallogr Commun. 2015 Aug 22;71(Pt 9):o682–3. doi: 10.1107/S2056989015015364 (PMC4555374; doi:10.1107/S2056989015015364)

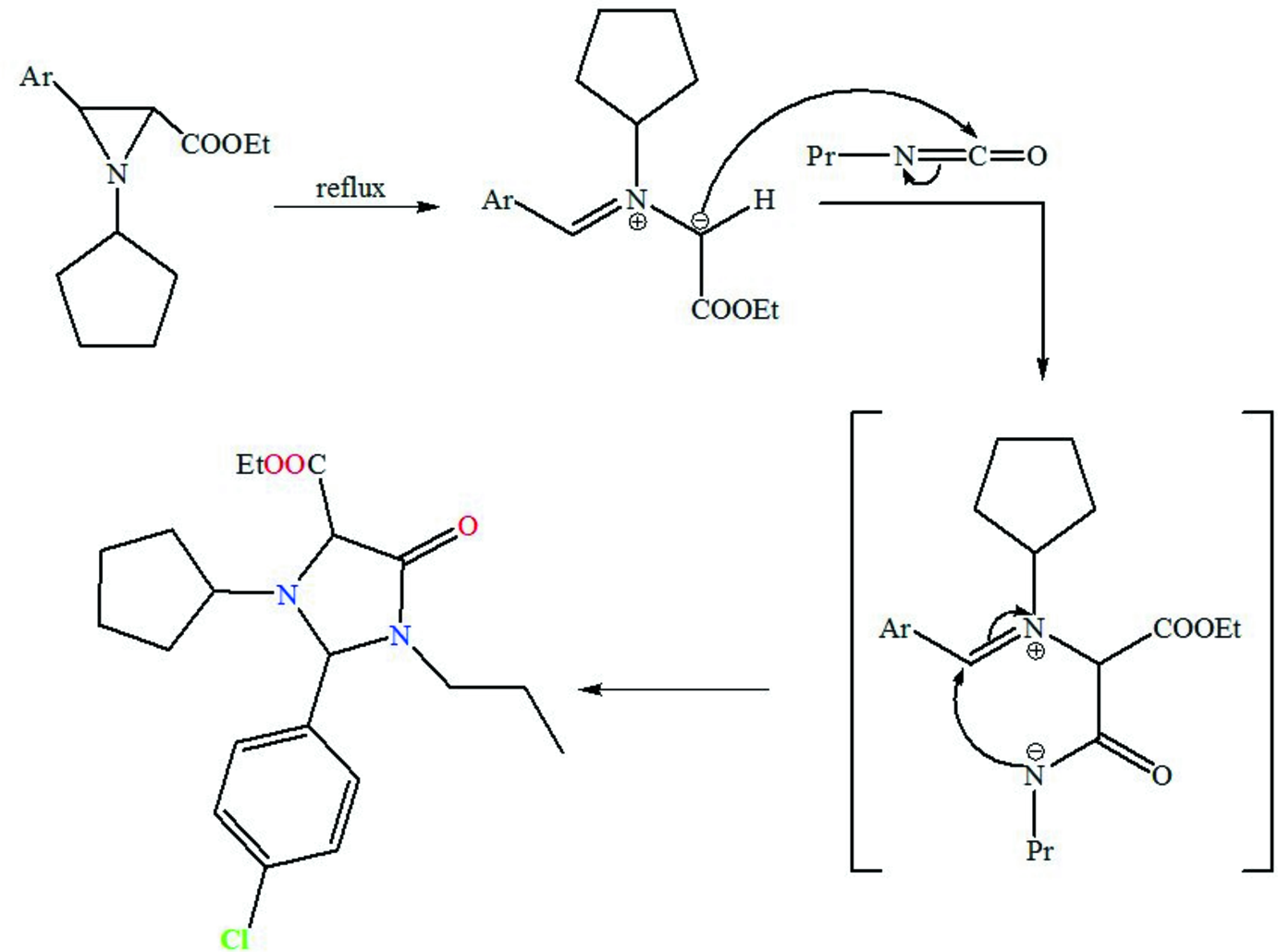

Supplement: Supplementary file 4 [file e-71-0o682-fig1.tif]

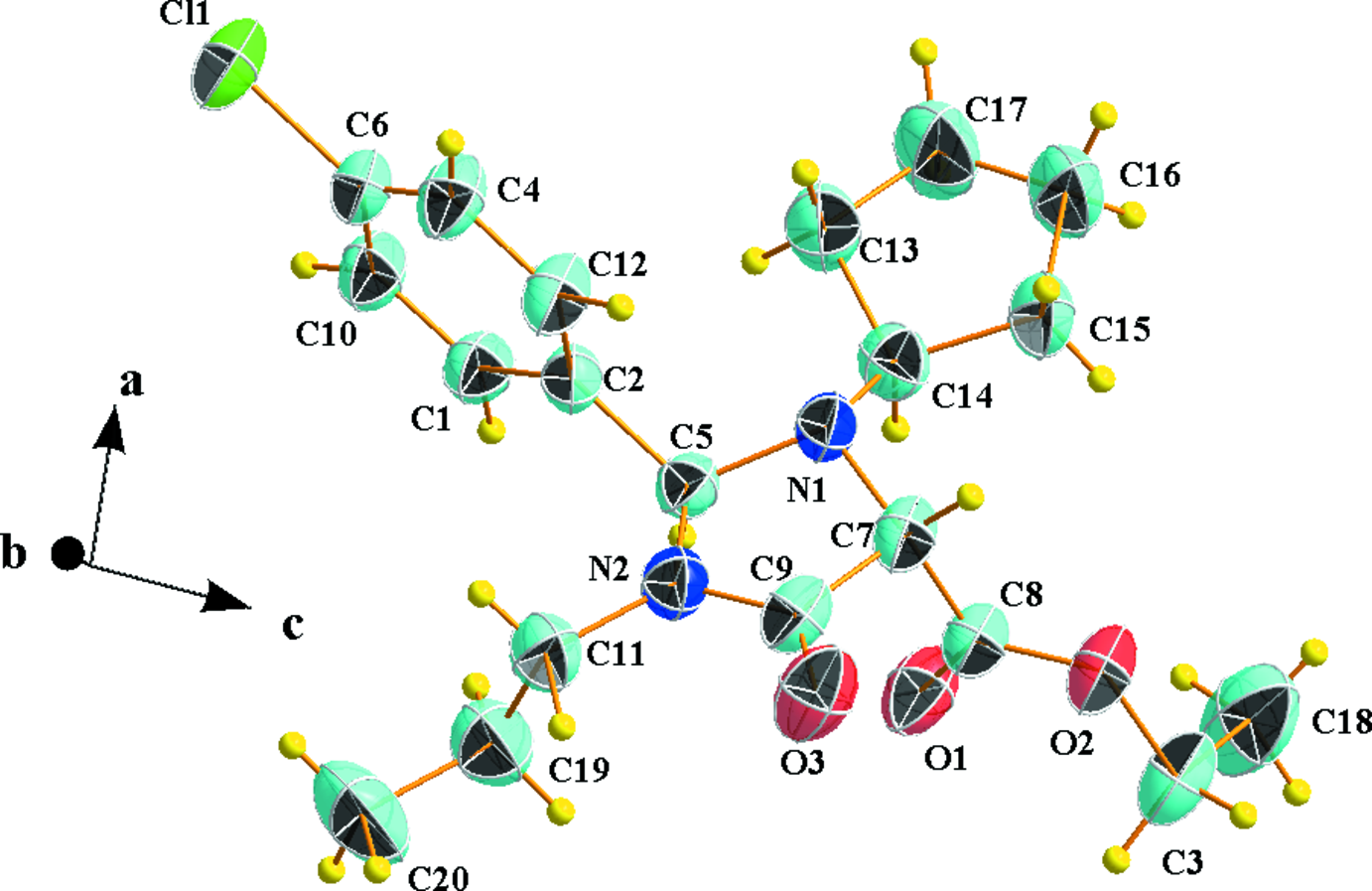

Supplement: Supplementary file 5 [file e-71-0o682-fig2.tif]

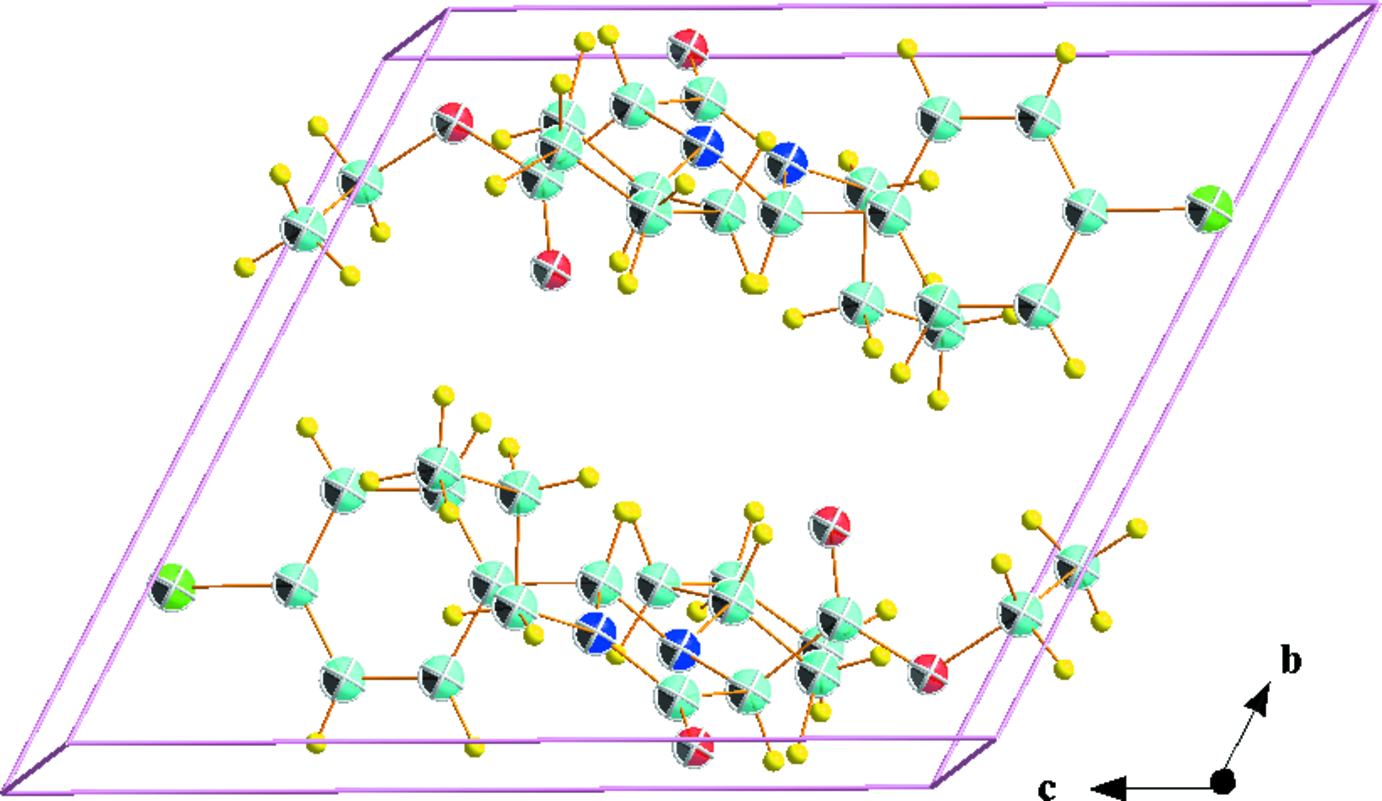

Supplement: Supplementary file 6 [file e-71-0o682-fig3.tif]
